# MRI data-driven clustering reveals different subtypes of Dementia with Lewy bodies

**DOI:** 10.1038/s41531-023-00448-6

**Published:** 2023-01-20

**Authors:** Anna Inguanzo, Konstantinos Poulakis, Rosaleena Mohanty, Christopher G. Schwarz, Scott A. Przybelski, Patricia Diaz-Galvan, Val J. Lowe, Bradley F. Boeve, Afina W. Lemstra, Marleen van de Beek, Wiesje van der Flier, Frederik Barkhof, Frederic Blanc, Paulo Loureiro de Sousa, Nathalie Philippi, Benjamin Cretin, Catherine Demuynck, Zuzana Nedelska, Jakub Hort, Barbara Segura, Carme Junque, Ketil Oppedal, Dag Aarsland, Eric Westman, Kejal Kantarci, Daniel Ferreira

**Affiliations:** 1 Division of Clinical Geriatrics, Center for Alzheimer Research, Department of Neurobiology, Care Sciences, and Society, Karolinska Institutet, Stockholm, Sweden; 2 Medical Psychology Unit, Institute of Neurosciences, University of Barcelona, Barcelona, Spain; 3 Institute of Biomedical Research August Pi i Sunyer (IDIBAPS), Barcelona, Spain; 4 Department of Radiology, Mayo Clinic, Rochester, MN US; 5 Quantitative Health Sciences, Mayo Clinic, Rochester, MN US; 6 Department of Neurology and Alzheimer Center, VU University Medical Center, Amsterdam, Netherlands; 7 UCL institutes of neurology and center for medical image computing, London, UK; 8 Day Hospital of Geriatrics, Memory Resource and Research Center (CM2R) of Strasbourg, Department of Geriatrics, Hopitaux Universitaires de Strasbourg, Strasbourg, France; 9 University of Strasbourg and French National Center for Scientific Research (CNRS), ICube Laboratory and Federation de Medecine Translationnelle de Strasbourg (FMTS), Team Imagerie Multimodale Integrative en Sante (IMIS)/ICONE, Strasbourg, France; 10 Department of Neurology, Charles University, 2nd Faculty of Medicine, Motol University Hospital, Prague, Czech Republic; 11 International Clinical Research Center, St. Annes University Hospital Brno, Brno, Czech Republic; 12 Center for Age-Related Medicine, Stavanger University Hospital, Stavanger, Norway; 13 Stavanger Medical Imaging Laboratory (SMIL), Department of Radiology, Stavanger University Hospital, Stavanger, Norway; 14 Department of Electrical Engineering and Computer Science, University of Stavanger, Stavanger, Norway; 15 Department of Neuroimaging, Center for Neuroimaging Sciences, Institute of Psychiatry, Psychology and Neuroscience, Kings College London, London, UK

**Keywords:** Dementia, Neurodegeneration

## Abstract

Dementia with Lewy bodies (DLB) is a neurodegenerative disorder with a wide heterogeneity of symptoms, which suggests the existence of different subtypes. We used data-driven analysis of magnetic resonance imaging (MRI) data to investigate DLB subtypes. We included 165 DLB from the Mayo Clinic and 3 centers from the European DLB consortium and performed a hierarchical cluster analysis to identify subtypes based on gray matter (GM) volumes. To characterize the subtypes, we used demographic and clinical data, as well as β-amyloid, tau, and cerebrovascular biomarkers at baseline, and cognitive decline over three years. We identified 3 subtypes: an older subtype with reduced cortical GM volumes, worse cognition, and faster cognitive decline (*n* = 49, 30%); a subtype with low GM volumes in fronto-occipital regions (*n* = 76, 46%); and a subtype of younger patients with the highest cortical GM volumes, proportionally lower GM volumes in basal ganglia and the highest frequency of cognitive fluctuations (*n* = 40, 24%). This study shows the existence of MRI subtypes in DLB, which may have implications for clinical workout, research, and therapeutic decisions.

## Introduction

Dementia with Lewy bodies (DLB) is a heterogeneous neurodegenerative disease in which alpha-synuclein is the main pathological hallmark. However, concomitant Alzheimer’s disease (AD) and cerebrovascular disease are common in DLB, contributing to disease heterogeneity^1–3^. Magnetic resonance imaging (MRI) has recently emerged as a promising technique to disentangle disease heterogeneity both in DLB^4^ and AD^5^. Early DLB studies focused on medial temporal areas as a key driver of clinical progression in DLB^6^. Recently, Oppedal & Ferreira et al. (2019)^4^, extended this approach to include posterior and frontal brain areas by classifying probable DLB patients into 4 brain atrophy subtypes previously described in AD^7^. However, these type of studies are still scarce and are all hypothesis-driven, while data-driven studies can reveal important aspects of the heterogeneity in neurodegenerative diseases^8^. For instance, in Parkinson’s disease (PD), another alpha-synuclein disease, data-driven studies have revealed that different brain atrophy subtypes explain part of the phenotype^9–11^.

The overall goal of this study was to advance our current understanding of the biological heterogeneity within DLB by using a data-driven clustering method applied on MRI. We identified DLB subtypes based on gray matter (GM) volumetric patterns and investigated whether these subtypes influence clinical phenotype, vary in the frequency of concomitant AD and cerebrovascular disease, and differ in cognitive trajectories over 3 years.

## Results

### Cohort characteristics

The cohort included 165 patients with probable DLB, 72% male, average age 69 years (SD = 8.57, range 45–88 years) and mean disease duration of 5.65 years (SD = 4.34). The mean years of education was 13.63 years (SD = 3.88). The mean Mini-Mental State Examination (MMSE) score was 22.91 (SD = 5.22), and the mean white matter hyperintensity (WMH) burden was 16.12 cm^3^ (SD = 13.25), which roughly corresponds to a Fazekas score^12^ of 2 (moderate WMH burden). Regarding the core clinical features, 55% of the patients had visual hallucinations (VH), 83% had cognitive fluctuations (CF), 87% had parkinsonism, and 78% had probable REM sleep behavior disorder (RBD). 43% of the patients were *APOE* ε4 carriers and 11% were classified as having concomitant AD (cerebrospinal fluid (CSF) subsample, *n* = 122).

### Data-driven analysis using random forest

The three-cluster solution showed the highest Calinski-Harabasz index (CH = 167.41), compared to the two-cluster solution (CH = 105.14) and the four-cluster solution (CH = 157.46). Combined with visual inspection of the dendrogram (Supplementary Fig. 1), we selected the three-cluster solution for subsequent analyses. The Random Forest (RF) proximity assessment showed robustness and stability of the three-cluster solution (Supplementary Fig. 2).

### Morphological characterization of the MRI subtypes

**Fig. 1:**
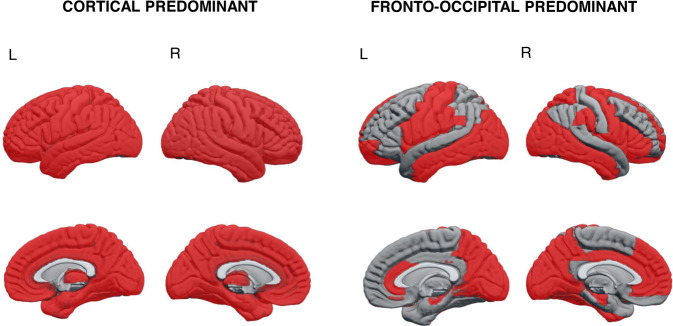
Patterns of gray matter atrophy in the cortical and fronto-occipital predominant subtypes. Z-scores adjusted by total ICV, center of origin and age were calculated using the subcortical predominant subtype as the group of reference. Red color depicts the ROIs in which the cortical and fronto-occipital predominant subtypes showed significantly reduced GM volumes with z-scores below −0.5, compared to the subcortical predominant subtype (see Supplementary Table 1 for ANCOVA results). ROI region of interest, ICV intracranial volume.

**Fig. 2:**
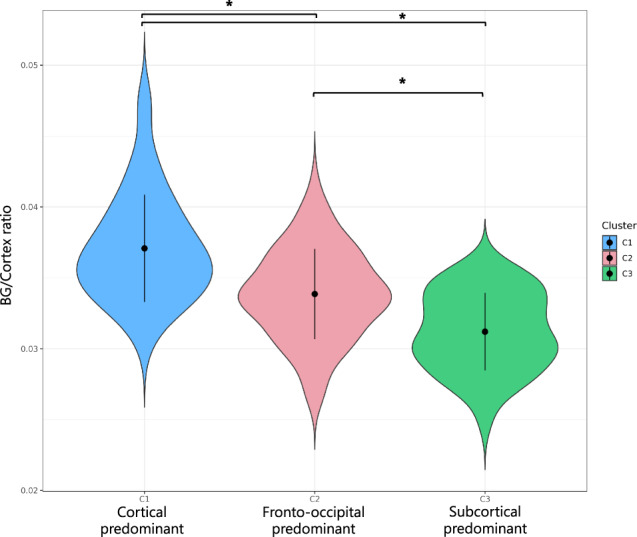
Ratio of basal ganglia to cortical GM volumes. The plot illustrates the distribution of the ratio across clusters. Significance for pair-wise comparisons is indicated with an asterisk (*p* < 0.05). BG basal ganglia, C1 cluster 1, C2 cluster 2, C3 cluster 3.

Whole-brain GM patterns were characterized by comparing the subtypes across the 96 regions of interest (ROIs) entered in the cluster analysis. ANCOVA results are shown in Supplementary Table 1, and Fig. 1 summarizes the regional differences between clusters. Cluster 1 (C1) was the subtype with overall lower GM volumes in cortical ROIs compared to cluster 2 (C2) and 3 (C3) (Supplementary Table 1). In consequence, we labeled C1 as the ‘cortical predominant’ subtype. C2 had intermediate GM volumes and showed prominent occipital and frontal differences particularly in medial and orbital frontal areas, as compared to C3 (Fig. 1). Hence, we labeled C2 as the ‘fronto-occipital predominant’ subtype. Finally, C3 had the highest cortical GM volumes but did not differ in the volume of basal ganglia (BG) GM with the other two clusters. Therefore, C3 was labeled as the ‘subcortical predominant’ subtype. To further investigate BG volumes in relation to cortical volumes, we computed a ratio by adding the bilateral volumes of the pallidum, putamen and caudate, and dividing them by the total sum of all cortical ROIs (Fig. 2). The ratio was significantly lower in C3 (x̄ = 0.031, SD = 0.003) than in C1 (x̄ = 0.037, SD = 0.004, 95%CI 0.004–0.008, *p* < 0.001), and C2 (x̄ = 0.034, SD = 0.003, 95%CI 0.001–0.004, *p* < 0.001). The ratio was also significantly lower in C2 than in C1 (95%CI 0.002–0.005, *p* < 0.001). Altogether, the ratio results support the finding that C1 is cortical predominant and C3 is subcortical predominant, with C2 in between.

**Fig. 3:**
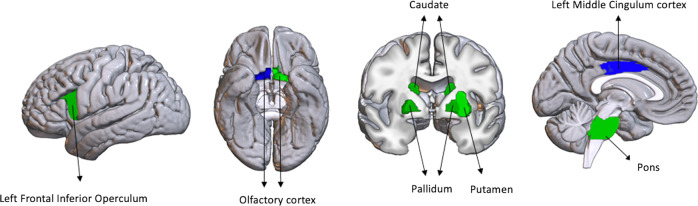
Visualization of the 10 most relevant regions in discriminating the 3 clusters. The supervised random forest model performed with the 10 most relevant ROIs showed that (in blue) the left middle cingulum discriminated the cortical predominant (C1) and fronto-occipital (C2) subtypes from the subcortical predominant (C3) subtype, while the right olfactory cortex discriminated the cortical predominant (C1) subtype from the fronto-occipital (C2) subtype.

Figure 3 shows the 10 most important ROIs in discriminating the 3 clusters (Supplementary Table 2 and Supplementary Fig. 3). GM volumes in the left middle cingulum and right olfactory cortex were the most relevant in discriminating the clusters.

### Clinical characterization of the MRI subtypes

C3 included the youngest patients and C1 the oldest ones (Table 1). C1 had higher years of education than both C2 and C3, and had significantly worse MMSE scores at baseline compared with C3. The differences in MMSE remained after accounting for age and education (F(2, 161) = 5.936, *p* = 0.005).

**Table 1:** Demographic, clinical, and biomarker characteristics of the DLB clusters.

	C1 (*N* = 49) Cortical predominant	C2 (*N* = 76) Fronto-occipital predominant	C3 (*N* = 40) Subcortical predominant	statistic (*p*-value)	post-hoc	Number of subjects with available data
Age, mean (SD)	73.43 (8.02)	69.05(7.52)	63.68(8.23)	17.048 (<0.001)	C1 > C2 C1 > C3 C2 > C3	C1:49 C2:76 C3:40
Years of education, mean (SD)	15.02(3.61)	13.24(3.90)	12.60 (3.80)	5.117 (0.007)	C1 > C2 C1 > C3	C1:49 C2:76 C3:40
MMSE, mean (SD)	21.57(5.49)	22.93(5.27)	24.50(4.38)	3.579 (0.030)	C3 > C1	C1:49 C2:75 C3:40
Sex, male/female (male %)	35/14 (71%)	60/16 (79%)	24/16 (60%)	4.696 (0.096)		C1:49 C2:76 C3:40
Disease duration (years), mean (SD)	5.11 (3.63)	5.09 (3.46)	7.25 (6.00)	2.961 (0.056)		C1:32 C2:59 C3:32
Visual hallucinations (presence/absence)	31/16 (66%)	39/36 (52%)	19/21 (48%)	3.461 (0.177)		C1:47 C2:75 C3:40
Cognitive fluctuations (presence/absence)	32/13 (71%)	63/11 (85%)	36/2 (95%)	8.614(0.013)	C3 > C1	C1:45 C2:74 C3:38
Probable RBD (presence/absence)	35/10 (78%)	57/13 (81%)	25/10 (71%)	1.362 (0.506)		C1:45 C2:70 C3:35
Parkinsonism (presence/absence)	43/4 (94%)	68/8 (90%)	31/9 (78%)	4.473(0.107)		C1:47 C2:76 C3:40
APOE genotype, ε4 carriers (presence/absence)	18/31 (37%)	38/34 (53%)	13/25 (34%)	4.770(0.092)		C1:49 C2:72 C3:38
AD co-pathology (presence)	7%	15%	6%	2.352 (0.309)		C1:29 C2:60 C3:33
White matter hyperintensities, mean (SD)	21.37 (15.41)	14.55 (13.13)	12.43(7.96)	6.230 (0.002)	C1 > C2 C1 > C3	C1:49 C2:76 C3:40
Center of origin (Mayo Clinic/ Prague/ Strasbourg/ Amsterdam)	28/14/4/3	26/12/15/23	14/3/15/8	27.800 (*p* < 0.001)	C1 vs C3 C1 vs C2	C1:49 C2:76 C3:40

*AD* Alzheimer’s disease, *C* Cluster, *CI* Confidence Interval, *DLB* Dementia with Lewy bodies, *MMSE* Mini Mental State Examination, *RBD* Rapid-eye movement behavior disorder, *SD* Standard deviation, One-way ANOVA was used for continuous variables, and the chi-squared test for categorical variables.

**Fig. 4:**
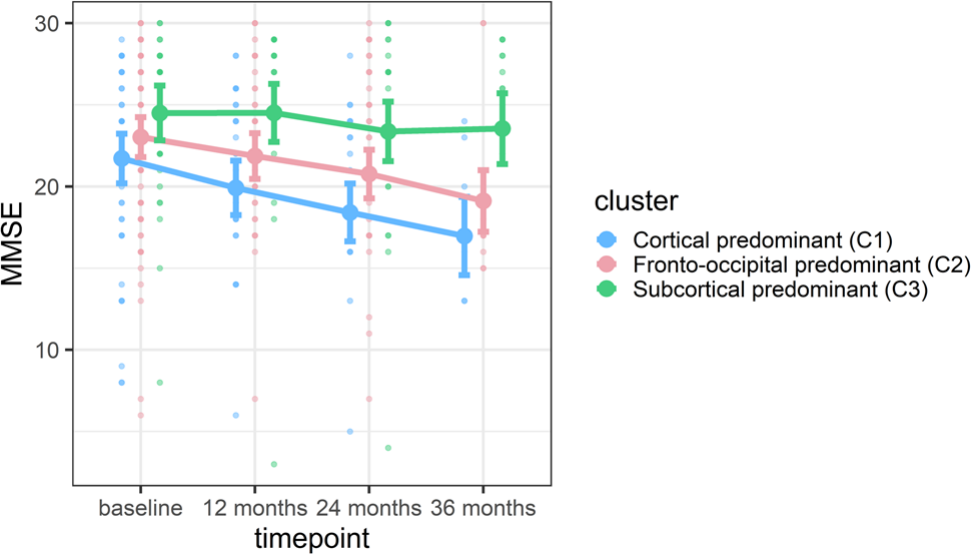
Cognitive decline over 3 years of follow-up as measured with the MMSE. Dots represent raw data in the background, the darker dots involve several individuals with the same score. Lines in the foreground represent estimated marginal means and error bars based on the standard error obtained from the linear mixed model. The cortical predominant subtype (C1) had significantly lower MMSE scores than the subcortical predominant subtype (C3) at baseline (*p* = 0.042), with increasing magnitude of the differences over time as reflected by the lower MMSE scores at 12-month follow-up (*p* < 0.001) the 24-month follow-up (*p* < 0.001) and the 36-month follow-up (*p* < 0.001). The fronto-occipital (C2) subtype had lower MMSE scores than the subcortical predominant (C3) subtype at the 36-month follow-up (*p* = 0.007). The cortical predominant (C1) and fronto-occipital (C2) subtypes did not differ in MMSE scores over time. At baseline, MMSE scores were available for 49 (C1), 75 (C2), and 40 (C3) DLB patients; at the 12-month follow-up, MMSE scores were available for 30 (C1), 38 (C2), and 29 (C3) DLB patients; at the 24-month follow-up, MMSE scores were available for 22(C1), 29 (C2), and 25 (C3) DLB patients; and at the 36-month follow-up MMSE scores were available for 7 (C1), 12 (C2), and 11 (C3) DLB patients. C1 Cluster 1 (cortical predominant subtype), C2 Cluster 2 (fronto-occipital predominant subtype), C3 Cluster 3 (subcortical predominant subtype), MMSE Mini-Mental State Examination.

C1 had a higher WMH burden than the other 2 subtypes (Table 1), but the differences disappeared after statistically controlling for age (F(2, 162) = 2.643, *p* = 0.074). The longitudinal analysis of MMSE trajectories over 3 years showed that C1 had a more rapid cognitive decline than C3 (Fig. 4, Supplementary Table 3 and Supplementary Table 4). When adjusting the longitudinal MMSE analysis for WMH, WMH did not have any significant effect in the prediction of MMSE trajectories and results remained the same, with C1 having a more rapid cognitive decline than C3 (please see Supplementary Tables 3 and 4).

Regarding the core clinical features, C3 showed a significantly higher frequency of CF (95%) compared with C1 (71%) (*p* = 0.013) (Table 1). There were no significant differences in the other core clinical features (Table 1). The frequency of *APOE* ε4 carriers was statistically comparable across groups. Patients from all four centers were evenly distributed across the 3 clusters.

## Discussion

In this study, we expanded previous hypothesis-driven MRI subtyping studies in DLB by conducting a data-driven MRI subtyping. We included a relatively large multi-center cohort including countries from Europe and the US. We found 3 subtypes within DLB: (1) a cortical predominant subtype, which included older patients with lower GM volumes and worse global cognition; (2) a fronto-occipital predominant subtype with intermediate GM volumes; and (3) a subcortical predominant subtype, which included younger patients with higher GM volumes and a higher frequency of CF. Differences in GM volumes and global cognition were independent of age.

The cortical predominant subtype had the lowest GM volumes across all cortical regions, as well as the worst global cognitive performance. This subtype resembles the widespread cortical atrophy subtype with worst cognitive performance previously reported in PD^11^. A similar widespread cortical atrophy subtype in AD has also been described to be the subtype with worst cognitive performance and fastest cognitive decline^5^. The fronto-occipital predominant subtype had intermediate age and intermediate GM volumes across many of the cortical regions, with particular involvement of frontal and occipital regions. This pattern resembles the fronto-occipital subtype described in PD^9^. The subcortical predominant subtype had the highest GM volumes, which is reminiscent of the minimal-atrophy subtype described in Oppedal & Ferreira et al. (2019)^4^. Similarly, cluster analyses in PD have repeatedly found a subtype with minimal or no atrophy at all^9,11^. Despite the prominent differences in cortical GM volumes, we did not find any significant differences in the volume of BG GM across subtypes, being the subcortical predominant subtype the one with proportionally lower volumes in BG compared to the other 2 subtypes. This finding may indicate the same level of atrophy in the BG in our three subtypes, as DLB patients have on average reduced GM volumes in BG compared to healthy controls (HC)^13–15^. Reduced GM volumes in BG have been associated with attentional deficits in DLB, suggesting that BG may be an early site of neurodegeneration^16^.

Altogether, our 3 subtypes showed an overall gradient of neurodegeneration with low GM volumes in the cortical predominant subtype, intermediate GM volumes in the fronto-occipital subtype, and highest GM volumes in the subcortical predominant subtype. An important question is whether our DLB subtypes reflect different stages of the disease or distinct subtypes. The cortical predominant subtype could represent DLB patients at a more advanced stage while fronto-occipital and subcortical predominant subtypes could represent less advanced stages. However, the different morphological patterns and the lower cognitive performance in cortical predominant remained after the statistical control for age, which suggests that cortical predominant may represent a subtype with a more aggressive progression. This interpretation is supported by two findings. Firstly, cortical predominant showed the most rapid cognitive decline over 3 years, while subcortical predominant had relatively stable cognitive performance over time. Secondly, there were no significant differences in disease duration across subtypes, which supports the hypothesis that our clusters reflect different subtypes rather than different disease stages. In fact, the subcortical predominant subtype had higher GM volumes but had, qualitatively, the longest disease duration. Hence, the late-onset form of DLB seems to confer a more aggressive presentation, while the early-onset form seems to have a better prognosis, as it has previously been described in PD^17^. In other diseases such as AD, the cortical predominant subtype is also a more aggressive presentation and is currently considered as a distinct subtype rather than a disease stage^5,18,19^. In addition, the differences in clinical features described below further support this interpretation on different subtypes rather than subgroups at different stages of the disease.

Clinically, the 3 subtypes only differed significantly in the presence of CF. Even though subcortical predominant was the subtype with highest cortical GM volumes, it was also the subtype with the highest frequency of patients with CF. CF have been related to altered functional connectivity of subcortical regions such as the pallidum and the putamen with the fronto-parietal network^20^. This finding could explain the higher frequency of CF in subcortical predominant, a subtype that has proportionally lower GM volumes in the BG. The dynamic nature of CF could be related to disconnection between cortical and subcortical GM structures in the subcortical predominant subtype. Brain disconnection has been suggested as one of the explanations for the minimal atrophy subtype of AD^21^, a subtype that has the highest cortical GM volumes, like our subcortical predominant subtype. It is also worth noting that the subcortical predominant subtype had the highest global cognitive performance at baseline and over time. The different atrophy patterns, together with the different cognitive trajectories, suggest that characterizing neuropsychological profiles, in these subtypes, could help to further elucidate their distinct nature by revealing different cognitive signatures as previously done in PD^9–11^ and AD^5^. In AD, for example, the hippocampal sparing subtype is known to have less memory impairment but more visual deficits than the other subtypes^5^.

Concerning VH, despite no statistically significant differences between subtypes, visual inspection suggested that the cortical predominant subtype had the highest frequency (66%) of VH, especially when compared with the subcortical predominant subtype (48%). Previous studies reported that patients with probable DLB and VH had reduced GM volumes in inferior frontal regions^22,23^ and cuneus^24^, when compared with patients with probable DLB without VH. In our study, the cortical predominant subtype had lower GM volumes in inferior frontal regions and cuneus when compared with both fronto-occipital and subcortical predominant subtypes.

Regarding parkinsonism, the groups did not differ in this clinical feature, which could be explained by comparable absolute GM volumes in BG across subtypes. Dysfunction of the BG is a well-known hallmark of DLB^25^ and is often related with motor impairment. For example, aberrant functional connectivity of the BG has been described in diseases with motor impairment such as PD^26^ and is independent of cognitive status in PD^27^. Another example is the association between motor impairment and alterations in a BG network both in PD^28^ and DLB^29^. However, we acknowledge that medications may influence motor symptoms^30^, as well as functional connectivity in the brain. Hence, future studies looking at structural MRI and functional connectivity of BG in relation to medications and parkinsonism across DLB subtypes are of interest, and may contribute to advance our understanding of the heterogeneity within DLB.

In addition, comorbid brain pathologies could be one of the factors contributing to MRI subtypes in DLB. We found a higher WMH burden (proxy for cerebrovascular disease) in cortical predominant compared to the other two subtypes, which seemed to be primarily explained by the older age of this subtype. Nonetheless, a recent study using the same sample than in our current study demonstrated that the WMH findings go beyond the mere effect of increasing age in DLB^2^. In addition, WMH burden influenced clinical phenotype as reflected in the association of a higher WMH burden with a higher frequency of visual hallucinations and lower MMSE scores^2^. Additionally, in that previous study, WMHs were associated with GM degeneration in several cortical areas characteristic of the cortical predominant subtype, particularly, the olfactory cortex^2^. Hence, despite that we observed that the WMH differences across subtypes disappeared when controlling for age, we cannot completely exclude that WMH burden may contribute somehow to our subtypes. We investigated global WMH, but regional WMH or other markers of cerebrovascular disease could help confirming this hypothesis. In contrast to WMH burden, the frequency of AD co-pathology (positive β-amyloid and tau biomarkers) did not reflect the age differences found in our subtypes, despite AD co-pathology increases with age in DLB^31^. Rather, visual inspection suggests that the fronto-occipital predominant subtype had the highest frequency of AD co-pathology, which is supported by the tendency to include a higher frequency (53%) of *APOE* ε4 carriers than the cortical (37%) and subcortical (34%) predominant subtypes, since *APOE* ε4 is the strongest genetic risk factor for AD^32^. Further, the pattern of amyloid PET binding in DLB with AD co-pathology^33^ includes very similar cortical areas to those describing our fronto-occipital predominant subtype.

Hippocampal volume has traditionally been regarded as a proxy of AD pathology, and explains part of the heterogeneity in the clinical phenotype of DLB^6,34,35^. Contrarily, in our study, by using a data-driven method to identify MRI subtypes of DLB, we found that hippocampal volume was not among the regions that best reflected the heterogeneity in GM patterns. A possible explanation is that hippocampal volume is usually spared in DLB^25^; in consequence, hippocampal volume may only acquire a relevant role at advanced stages of DLB, where AD co-pathology is higher^1^, as in the cohorts often included in postmortem studies. Contrarily, at less advanced stages of the disease, the presence of AD co-pathology is lower^31^, as reflected by the low proportion of DLB patients with positive AD biomarkers in our study.

The current study has some limitations. Firstly, we did not have a HC group. However, previous studies from the centers included in our current study show that our DLB patients do have brain atrophy when compared with a HC group^15,36,37^. Although our main goal was to identify and characterize MRI subtypes in DLB, having a HC group could help to further describe some aspects of our subtypes. Secondly, we had some missing data for β-amyloid and tau biomarkers, giving a smaller subsample for statistical analysis. Still, we reported the proportion of biomarker-positive DLB patients along with group sizes due to the clinical interest of those data. Thirdly, clustering in the current study is cross-sectional, like virtually all current MRI subtyping studies^5,8^. The advent of new longitudinal clustering methods^38^ will open the door for future longitudinal subtyping studies in DLB, helping to better characterize disease progression of our current DLB subtypes.

In conclusion, by using a data-driven approach on a relatively large cohort of probable DLB patients, we found 3 MRI subtypes characterized by different patterns of GM volumes and clinical profiles. Our approach has been demonstrated to be useful in DLB, and we hope it can inspire future works to help establish distinct neurodegeneration subtypes, as well as their links with close disorders such as PD and AD. The ultimate goal would be to leverage this knowledge to realize personalized medicine approaches, in which biomarkers and subtypes would guide therapeutic decisions in neurodegenerative diseases.

## Methods

### Participants

The data of this multicenter study were a combination of the European DLB consortium (E-DLB) (*n* = 97)^39^, including 29 patients from Prague, 34 from Strasbourg, and 34 from Vumc Amsterdam, and the Mayo Clinic DLB cohort from Rochester, MN, United States (*n* = 68), making a total of 165 DLB patients. Diagnosis and core clinical features (parkinsonism, VH, CF and RBD) were based on the 2005 International Consensus Criteria for probable DLB^40^. Core clinical features were assessed with well-established instruments (please see https://www.e-dlb.com/psychosometric-and-clinical-measurements/), but due to the multi-center nature of this study, outcomes were dichotomized as presence/absence of features for harmonization and statistical analyses in the current study. As a measure of global cognition, we used the MMSE assessed annually over 3 years. Exclusion criteria were: (i) presence of acute delirium, (ii) terminal illness, (iii) previous stroke, (iv) psychotic or bipolar disorder, (v) craniocerebral trauma, and (vi) recent diagnosis of a major somatic illness. Local ethics committee at each E-DLB center and the Mayo Clinic Institutional Review Board approved the study. Written consent on participation was obtained from all patients or appropriate surrogates according to the Declaration of Helsinki.

### β-amyloid and tau biomarkers

β-amyloid and tau pathologies were assessed for a total of 122 DLB patients with CSF β-amyloid 1-42 and phosphorylated tau biomarkers in the E-DLB cohort, and with positron emission tomography (PET) Pittsburgh compound B (PiB) and Flortaucipir (AV-1451) tracers in the Mayo Clinic. Biomarker levels were classified as normal or abnormal based on center-specific established cut points, as explained in previous studies^31,41^. AD co-pathology was defined as positivity in both β-amyloid and tau biomarkers.

### Neuroimaging data

A high-resolution 3D T1-weigthed magnetization prepared rapid gradient echo (MPRAGE) sequence and a FLAIR sequence were acquired in 3T (The Day Hospital of Geriatrics, Memory Resource and Research Center, CMRR, Strasbourg, France; the VU University Medical Center, Vumc, Amsterdam, the Netherlands; and the Mayo Clinic, Rochester, US) and 1.5T (Motol University Hospital, Prague, Czech Republic) scanners.

Images from the E-DLB consortium were managed through the hive database system (theHiveDB)^42^. All the data was preprocessed at the Mayo Clinic. GM volumes from 82 cortical ROIs, 12 subcortical ROIs, and 2 brainstem ROIs (Supplementary Table 5) were obtained using the Mayo Clinic Adult Lifespan Template (MCALT) (https://www.nitrc.org/projects/mcalt/) atlas. Analyses were carried out with the residuals obtained from a multiple linear regression model where each ROI was adjusted for center and ICV. Cerebrovascular disease was assessed through WMH burden on FLAIR, using a semi-quantitative method described in previous publications^2^.

### Subtypes of DLB based on data-driven analysis

We performed a cluster analysis with the RF method applied on the residuals of the 96 volumetric ROIs^43^. The RF method provided a similarity matrix that was then used as the input for the agglomerative hierarchical clustering using the average linkage method^44^. The Calinski-Harabasz index was used to evaluate the optimal number of clusters, where 2 to 10 clusters were considered. The mean decrease in the Gini index was used to identify the ROIs with the highest contribution to the cluster analysis. The 10 most relevant ROIs were then used for a supervised RF model, in which the ROIs were the predictor variables and the cluster number the dependent variable. This supervised model was performed to identify the ROIs that best discriminated between the clusters.

To carefully test the robustness and stability of our cluster analysis, we carried out a RF proximity matrix assessment: we repeated the RF 100 times and computed the difference between the similarity matrix used in the main analysis and each of the 100 simulated similarity matrices^45^.

### Statistical analyses

Differences in demographics, clinical measures, and biomarkers were assessed with one-way ANOVA for continuous variables and the Pearson’s chi-squared test for categorical variables. Differences between clusters in GM across ROIs were assessed with ANCOVA adjusting by age. These analyses were performed using IBM SPSS Statistics 27.0 (IBM Corp., Armonk, New York). The results from ANCOVA were corrected for multiple comparisons using the false-discovery rate (FDR) adjustment across the 96 ROIs, with the significance level set at *p* < 0.05. Tests were 2-sided. R was used to implement RF and clustering analyses, as well as to assess cognitive trajectories over 3 years (MMSE scores) with a linear mixed model (LMM) (see Supplementary Methods 1).

### Reporting Summary

Further information on research design is available in the Nature Research Reporting Summary linked to this article.

## Supplementary information

Supplementary material

Reporting summary

## Data Availability

All data used for this study is available through the E-DLB consortium (https://www.e-dlb.com) and the Mayo Clinic (https://www.mayo.edu/research/labs/aging-dementia-imaging/overview) for qualified researchers upon request.
