# Supplementary material for: MRI data-driven clustering reveals different subtypes of Dementia with Lewy bodies
Source: NPJ Parkinsons Dis. 2023 Jan 20;9:5. doi: 10.1038/s41531-023-00448-6 (PMC9859778; doi:10.1038/s41531-023-00448-6)
Supplement: Supplementary file 1 — Supplementary material [file 41531_2023_448_MOESM1_ESM.pdf]

SUPPLEMENTARY MATERIAL

Supplementary Figure 1. Dendrogram from the cluster analysis.
Supplementary Figure 2. Random forest proximity matrix assessment.
Supplementary Table 1. Regional differences between clusters.
Supplementary Table 2. ROIs with the highest contribution to the cluster analysis.
Supplementary Figure 3. Supervised classification tree.
Supplementary Table 3. Longitudinal analysis of MMSE trajectories.
Supplementary Table 4. Longitudinal analysis of MMSE trajectories: post-hoc comparisons.
Supplementary Table 5. List of ROIs used in the cluster analysis.
Supplementary Methods 1. Longitudinal analysis of MMSE trajectories.

Supplementary Figure 1. Dendrogram from the cluster analysis.

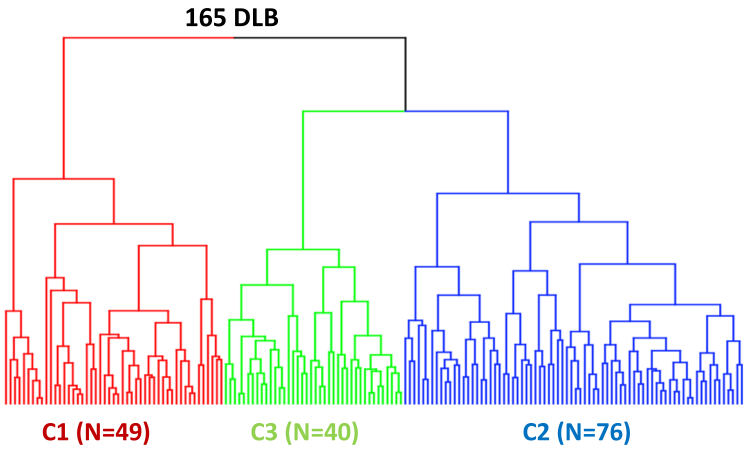

Dendrogram of the 165 patients with pDLB clustered according to gray matter volumes. Abbreviations: C1 – Cluster 1, C2 – Cluster 2, C3 – Cluster 3, DLB – Dementia with Lewy bodies.

Supplementary Figure 2. Random forest proximity matrix assessment.

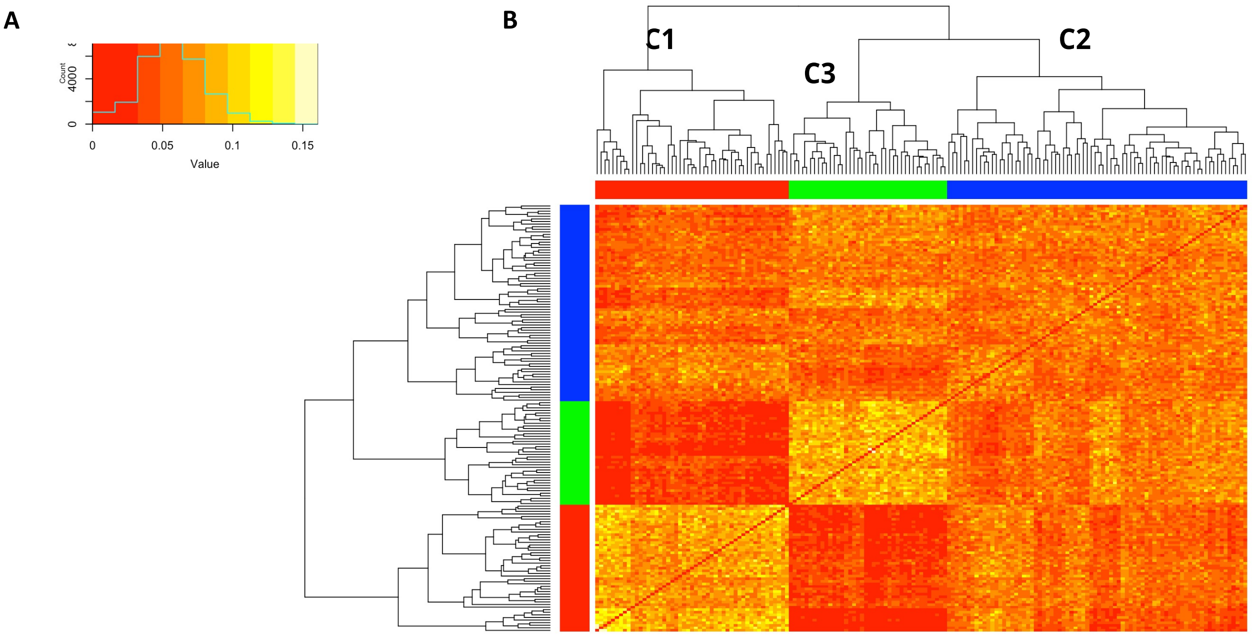

(A) Histogram with the colour key: values closer to 0 indicate non-similar observations while values closer to 1 indicate identical observations. Pairwise similarities shown in red are the most stable, while similarities shown in yellow are the least stable. Hence, findings are stable when values are closer to 0. (B) Matrix showing the average difference between the similarity matrix used as the input of the cluster analysis (the main analysis) and the similarity matrices obtained from the simulated 100 random forest models. The clustering dendrogram is displayed in the horizontal and vertical axes. Abbreviations: C1 – Cluster 1 (cortical predominant subtype); C2 - Cluster 2 (fronto-occipital predominant subtype); C3 – Cluster 3 (subcortical predominant subtype).

Supplementary Table 1. Regional differences between clusters.

	C1 (n=49)	C2 (n=76)	C3 (n=40)	C1 z-scores	C2 z-scores	F ANCOVA	FDR adjusted p-values	post-hoc paired test
**Precentral L**	-0.717 (1.058)	0.081 (1.156)	0.723 (0.972)	-1.4814	-0.6598	24.943	1.37E-09	C1 < C2<br>C1 < C3<br>C2 < C3
**Precentral R**	-0.613 (1.366)	0.038 (1.032)	0.679 (1.134)	-1.1395	-0.5652	17.002	3.56E-07	C1 < C2<br>C1 < C3<br>C2 < C3
**Frontal Sup L**	-0.471 (0.944)	0.028 (0.983)	0.522 (1.024)	-0.9707	-0.4821	14.044	3.42E-06	C1 < C2<br>C1 < C3<br>C2 < C3
**Frontal Sup R**	-0.365 (0.995)	-0.138 (1.033)	0.709 (0.875)	-1.2271	-0.968	17.731	2.11E-07	C1 < C3<br>C2 < C3
**Frontal Sup Orb L**	-0.259 (0.307)	0.017 (0.298)	0.283 (0.262)	-2.0651	-1.0107	50.636	1.19E-16	C1 < C2<br>C1 < C3<br>C2 < C3
**Frontal Sup Orb R**	-0.277 (0.328)	0.027 (0.310)	0.288 (0.384)	-1.4728	-0.6797	41.743	1.98E-14	C1 < C2<br>C1 < C3<br>C2 < C3
**Frontal Mid L**	-0.666 (1.946)	-0.053 (1.940)	0.917 (2.352)	-0.6733	-0.4125	8.104	0.000513	C1 < C3<br>C2 < C3
**Frontal Mid R**	-0.858 (1.914)	0.076 (1.913)	0.906 (1.961)	-0.9001	-0.423	11.519	2.70E-05	C1 < C2<br>C1 < C3<br>C2 < C3
**Frontal Mid Orb L**	-0.381 (0.440)	0.028 (0.463)	0.412 (0.494)	-1.6066	-0.7763	42.525	1.42E-14	C1 < C2<br>C1 < C3<br>C2 < C3
**Frontal Mid Orb R**	-0.382 (0.486)	0.104 (0.453)	0.270 (0.422)	-1.547	-0.3942	32.574	7.04E-12	C1 < C2<br>C1 < C3<br>C2 < C3
**Frontal Inf Oper L**	-0.169 (0.562)	-0.056 (0.529)	0.313 (0.629)	-0.7668	-0.5877	10.622	5.79E-05	C1 < C3<br>C2 < C3
**Frontal Inf Oper R**	-0.369 (0.432)	0.034 (0.457)	0.387 (0.635)	-1.192	-0.5554	32.956	5.65E-12	C1 < C2<br>C1 < C3<br>C2 < C3
**Frontal Inf Tri L**	-0.337 (0.576)	0.062 (0.537)	0.293 (0.575)	-1.0969	-0.4013	18.469	1.23E-07	C1 < C2<br>C1 < C3<br>C2 < C3
**Frontal Inf Tri R**	-0.328 (0.569)	0.003 (0.615)	0.395 (0.564)	-1.2838	-0.6958	20.859	2.22E-08	C1 < C2<br>C1 < C3<br>C2 < C3
**Frontal Inf Orb L**	-0.525 (0.664)	0.152 (0.527)	0.352 (0.510)	-1.7178	-0.3905	39.274	9.45E-14	C1 < C2<br>C1 < C3<br>C2 < C3
**Frontal Inf Orb R**	-0.462 (0.634)	0.094 (0.522)	0.387 (0.579)	-1.468	-0.5052	33.306	4.71E-12	C1 < C2<br>C1 < C3<br>C2 < C3
**Rolandic Oper L**	-0.282 (0.354)	-0.014 (0.348)	0.374 (0.418)	-1.5702	-0.9309	46.489	1.23E-15	C1 < C2<br>C1 < C3<br>C2 < C3

	C1 (n=49)	C2 (n=76)	C3 (n=40)	C1 z-scores	C2 z-scores	F ANCOVA	FDR adjusted p-values	post-hoc paired test
**Rolandic Oper R**	-0.352 (0.487)	0.011 (0.422)	0.409 (0.518)	-1.4695	-0.768	38.287	1.59E-13	C1 < C2 C1 < C3 C2 < C3
**Supp Motor Area L**	-0.502 (0.563)	0.110 (0.710)	0.406 (0.894)	-1.0154	-0.3307	23.672	3.01E-09	C1 < C2 C1 < C3 C2 < C3
**Supp Motor Area R**	-0.409 (0.627)	0.026 (0.886)	0.452 (0.902)	-0.9555	-0.4725	15.102	1.49E-06	C1 < C2 C1 < C3 C2 < C3
**Olfactory L**	-0.037 (0.096)	0.001 (0.089)	0.0438 (0.105)	-0.7712	-0.4028	9.873	0.00011	C1 < C2 C1 < C3 C2 < C3
**Olfactory R**	-0.043 (0.104)	0.001 (0.101)	0.051 (0.108)	-0.873	-0.4717	11.3	3.23E-05	C1 < C2 C1 < C3 C2 < C3
**Frontal Sup Medial L**	-0.345 (0.830)	-0.105 (0.839)	0.623 (0.923)	-1.0496	-0.7893	18.695	1.07E-07	C1 < C3 C2 < C3
**Frontal Sup Medial R**	-0.297 (0.962)	-0.051 (1.130)	0.462 (1.064)	-0.7147	-0.4832	6.999	0.001343	C1 < C3 C2 < C3
**Frontal Med Orb L**	-0.332 (0.372)	0.055 (0.379)	0.302 (0.300)	-2.1163	-0.8252	47.162	9.07E-16	C1 < C2 C1 < C3 C2 < C3
**Frontal Med Orb R**	-0.273 (0.445)	0.083 (0.438)	0.176 (0.297)	-1.5128	-0.3136	19.379	6.43E-08	C1 < C2 C1 < C3
**Rectus L**	-0.1553 (0.285)	0.037 (0.238)	0.118 (0.324)	-0.8434	-0.2477	14.799	1.86E-06	C1 < C2 C1 < C3
**Rectus R**	-0.154 (0.241)	0.053 (0.251)	0.087 (0.265)	-0.9096	-0.1251	15.736	9.16E-07	C1 < C2 C1 < C3
**Insula L**	-0.454 (0.408)	0.043 (0.439)	0.473 (0.482)	-1.9224	-0.8898	67.639	1.14E-20	C1 < C2 C1 < C3 C2 < C3
**Insula R**	-0.534 (0.477)	0.109 (0.419)	0.447 (0.536)	-1.8311	-0.6311	71.318	6.34E-21	C1 < C2 C1 < C3 C2 < C3
**Cingulum Ant L**	-0.163 (0.508)	-0.052 (0.555)	0.300 (0.578)	-0.8026	-0.6107	10.378	7.09E-05	C1 < C3 C2 < C3
**Cingulum Ant R**	-0.241 (0.650)	-0.094 (0.806)	0.476 (0.662)	-1.0838	-0.8614	14.444	2.47E-06	C1 < C3 C2 < C3
**Cingulum Mid L**	-0.109 (0.384)	-0.039 (0.304)	0.209 (0.408)	-0.7801	-0.6098	11.748	2.24E-05	C1 < C3 C2 < C3
**Cingulum Mid R**	-0.219 (0.368)	0.026 (0.398)	0.217 (0.403)	-1.0806	-0.4711	17.428	2.60E-07	C1 < C2 C1 < C3 C2 < C3
**Hippocampus L**	-0.145 (0.354)	-0.010 (0.342)	0.199 (0.281)	-1.2253	-0.746	14.798	1.86E-06	C1 < C2 C1 < C3 C2 < C3
**Hippocampus R**	-0.152 (0.390)	0.011 (0.298)	0.165 (0.256)	-1.2364	-0.6001	13.513	5.23E-06	C1 < C2 C1 < C3 C2 < C3
**Amygdala L**	-0.034 (0.093)	0.001 (0.110)	0.040 (0.079)	-0.9404	-0.4915	7.723	0.001116	C1 < C3

	C1 (n=49)	C2 (n=76)	C3 (n=40)	C1 z-scores	C2 z-scores	F ANCOVA	FDR adjusted p-values	post-hoc paired test
**Amygdala R**	-0.059 (0.104)	0.010 (0.101)	0.051 (0.086)	-1.288	-0.4762	18.038	1.71E-07	C1 < C2<br>C1 < C3<br>C2 < C3
**Calcarine L**	-0.183 (0.814)	-0.188 (0.760)	0.582 (0.843)	-0.9075	-0.9136	16.618	4.80E-07	C1 < C3<br>C2 < C3
**Calcarine R**	-0.290 (0.757)	-0.082 (0.724)	0.512 (0.895)	-0.8958	-0.6639	15.334	1.26E-06	C1 < C3<br>C2 < C3
**Cuneus L**	-0.370 (0.7851)	-0.094 (0.742)	0.634 (0.917)	-1.0948	-0.7944	22.928	4.95E-09	C1 < C2<br>C1 < C3<br>C2 < C3
**Cuneus R**	-0.405 (0.708)	0.001 (0.718)	0.494 (0.848)	-1.0608	-0.5811	19.939	4.39E-08	C1 < C2<br>C1 < C3<br>C2 < C3
**Lingual L**	-0.547 (0.867)	0.008 (0.995)	0.655 (0.965)	-1.2462	-0.6701	22.166	8.75E-09	C1 < C2<br>C1 < C3<br>C2 < C3
**Lingual R**	-0.577 (0.837)	0.062 (0.797)	0.588 (0.895)	-1.3021	-0.5878	27.829	1.66E-10	C1 < C2<br>C1 < C3<br>C2 < C3
**Occipital Sup L**	-0.214 (0.477)	-0.134 (0.500)	0.518 (0.640)	-1.1437	-1.0194	31.469	1.39E-11	C1 < C3<br>C2 < C3
**Occipital Sup R**	-0.180 (0.719)	-0.082 (0.579)	0.377 (0.594)	-0.9387	-0.7727	11.969	1.87E-05	C1 < C3<br>C2 < C3
**Occipital Mid L**	-0.666 (1.158)	-0.062 (1.346)	0.934 (1.141)	-1.4024	-0.8734	23.167	4.20E-09	C1 < C2<br>C1 < C3<br>C2 < C3
**Occipital Mid R**	-0.494 (0.913)	-0.022 (0.770)	0.648 (0.819)	-1.3938	-0.8186	26.807	3.43E-10	C1 < C2<br>C1 < C3<br>C2 < C3
**Occipital Inf L**	-0.270 (0.574)	-0.097 (0.583)	0.517 (0.445)	-1.7683	-1.3804	31.237	1.55E-11	C1 < C2<br>C1 < C3<br>C2 < C3
**Occipital Inf R**	-0.338 (0.706)	-0.032 (0.670)	0.477 (0.629)	-1.2973	-0.8112	20.599	2.66E-08	C1 < C2<br>C1 < C3<br>C2 < C3
**Fusiform L**	-0.631 (0.988)	0.027 (0.977)	0.721 (0.795)	-1.7004	-0.872	29.204	6.26E-11	C1 < C2<br>C1 < C3<br>C2 < C3
**Fusiform R**	-0.726 (1.045)	0.160 (0.841)	0.584 (0.898)	-1.4588	-0.4713	30.798	2.06E-11	C1 < C2<br>C1 < C3<br>C2 < C3
**Postcentral L**	-0.667 (1.061)	0.021 (1.003)	0.777 (1.319)	-1.0952	-0.5732	23.889	2.63E-09	C1 < C2<br>C1 < C3<br>C2 < C3
**Postcentral R**	-0.553 (1.011)	0.061 (1.024)	0.560 (1.190)	-0.9353	-0.4187	15.241	1.34E-06	C1 < C2<br>C1 < C3<br>C2 < C3
**Parietal Sup L**	-0.482 (0.881)	0.090 (0.995)	0.419 (0.869)	-1.0374	-0.3782	13.364	5.86E-06	C1 < C2<br>C1 < C3
**Parietal Sup R**	-0.531 (0.819)	0.069 (0.774)	0.518 (0.857)	-1.2247	-0.5234	24.013	2.32E-09	C1 < C2<br>C1 < C3<br>C2 < C3

	C1 (n=49)	C2 (n=76)	C3 (n=40)	C1 z-scores	C2 z-scores	F ANCOVA	FDR adjusted p-values	post-hoc paired test
**Parietal Inf L**	-0.318 (0.450)	0.121 (0.423)	0.160 (0.468)	-1.0231	-0.0833	21.436	1.49E-08	C1 < C2 C1 < C3
**Parietal Inf R**	-0.370 (0.508)	0.064 (0.706)	0.332 (0.714)	-0.9839	-0.3746	16.386	5.71E-07	C1 < C2 C1 < C3 C2 < C3
**SupraMarginal L**	-0.489 (0.746)	0.061 (0.752)	0.482 (0.669)	-1.4526	-0.6287	25.151	1.15E-09	C1 < C2 C1 < C3 C2 < C3
**SupraMarginal R**	-0.391 (0.696)	0.023 (0.687)	0.433 (0.698)	-1.181	-0.5867	19.653	5.41E-08	C1 < C2 C1 < C3 C2 < C3
**Angular L**	-0.329 (0.757)	0.075 (1.044)	0.261 (0.983)	-0.6011	-0.1894	5.587	0.005393	C1 < C2 C1 < C3
**Angular R**	-0.649 (0.963)	0.117 (1.209)	0.572 (1.101)	-1.1095	-0.4131	17.247	2.96E-07	C1 < C2 C1 < C3 C2 < C3
**Precuneus L**	-0.938 (0.870)	0.054 (0.992)	1.045 (0.916)	-2.1652	-1.082	67.966	1.14E-20	C1 < C2, C1 < C3, C2 < C3
**Precuneus R**	-0.902 (0.986)	0.041 (0.971)	1.026 (1.100)	-1.753	-0.8955	54.214	1.60E-17	C1 < C2 C1 < C3 C2 < C3
**Paracentral Lobule L**	-0.359 (1.050)	0.058 (0.854)	0.328 (0.972)	-0.7072	-0.277	7.396	0.001116	C1 < C2 C1 < C3
**Paracentral Lobule R**	-0.305 (1.058)	0.012 (0.791)	0.351 (0.955)	-0.688	-0.3548	6.908	0.001116	C1 < C3
**Caudate L**	-0.0172 (0.335)	0.0001 (0.278)	0.021 (0.288)	-0.1331	-0.073	0.219	0.812463	n.s
**Caudate R**	-0.014 (0.387)	0.001 (0.324)	0.016 (0.317)	-0.0978	-0.05	0.109	0.897	n.s
**Pallidum L**	-0.001 (0.049)	0.004 (0.059)	-0.007 (0.059)	0.1091	0.2079	0.668	0.536348	n.s
**Pallidum R**	-0.002 (0.040)	0.004 (0.055)	-0.005 (0.056)	0.0521	0.1751	0.562	0.589419	n.s
**Putamen L**	-0.036 (0.408)	0.001 (0.463)	0.042 (0.445)	-0.1788	-0.0941	0.428	0.666894	n.s
**Putamen R**	-0.094 (0.415)	0.019 (0.442)	0.080 (0.384)	-0.4546	-0.1588	2.436	0.096	n.s
**Thalamus L**	-0.224 (0.288)	0.047 (0.247)	0.184 (0.322)	-1.2652	-0.4221	32.224	9.07E-21	C1 < C2 C1 < C3 C2 < C3
**Thalamus R**	-0.182 (0.311)	0.028 (0.272)	0.169 (0.334)	-1.0503	-0.4191	19.548	5.74E-08	C1 < C2 C1 < C3 C2 < C3
**Heschl L**	-0.158 (0.186)	0.028 (0.240)	0.138 (0.310)	-0.9548	-0.3528	21.207	1.74E-08	C1 < C2 C1 < C3 C2 < C3
**Heschl R**	-0.090 (0.146)	0.013 (0.170)	0.084 (0.220)	-0.7907	-0.32	13.532	5.22E-06	C1 < C2 C1 < C3 C2 < C3
**Temporal Sup L**	-0.744 (1.003)	0.240 (1.150)	0.456 (1.219)	-0.9851	-0.1772	18.815	9.91E-08	C1 < C2 C1 < C3

	C1 (n=49)	C2 (n=76)	C3 (n=40)	C1 z-scores	C2 z-scores	F ANCOVA	FDR adjusted p-values	post-hoc paired test
**Temporal Sup R**	-0.666 (0.995)	0.171 (1.056)	0.490 (1.218)	-0.9497	-0.262	17.501	2.50E-07	C1 < C2<br>C1 < C3
**Temporal Pole Sup L**	-0.216 (0.446)	0.048 (0.424)	0.174 (0.351)	-1.1124	-0.3588	12.995	7.92E-06	C1 < C2<br>C1 < C3
**Temporal Pole Sup R**	-0.232 (0.405)	0.057 (0.375)	0.176 (0.430)	-0.9525	-0.2783	15.902	8.11E-07	C1 < C2<br>C1 < C3
**Temporal Mid L**	-0.759 (1.385)	-0.152 (1.295)	1.219 (1.356)	-1.4601	-1.0118	31.992	1.01E-11	C1 < C2<br>C1 < C3<br>C2 < C3
**Temporal Mid R**	-1.217 (1.566)	0.1202 (1.505)	1.262 (1.477)	-1.6794	-0.7736	38.769	1.23E-13	C1 < C2<br>C1 < C3<br>C2 < C3
**Temporal Pole Mid L**	-0.217 (0.404)	0.017 (0.512)	0.234 (0.474)	-0.9531	-0.4579	12.478	1.22E-05	C1 < C2<br>C1 < C3<br>C2 < C3
**Temporal Pole Mid R**	-0.338 (0.440)	0.048 (0.502)	0.322 (0.567)	-1.1643	-0.4826	24.79	1.37E-09	C1 < C2<br>C1 < C3<br>C2 < C3
**Temporal Inf L**	-0.842 (1.073)	0.077 (0.990)	0.885 (0.994)	-1.7377	-0.8131	42.058	1.76E-14	C1 < C2<br>C1 < C3<br>C2 < C3
**Temporal Inf R**	-1.166 (1.040)	0.132 (1.144)	1.177 (1.213)	-1.9321	-0.8613	65.704	2.63E-20	C1 < C2<br>C1 < C3<br>C2 < C3
**Pons**	-0.013 (0.030)	-0.001 (0.027)	0.018 (0.063)	-0.5069	-0.3069	8.84	0.00027	C1 < C3<br>C2 < C3
**Dorsal Mesopontine**	-0.002 (0.007)	- 0.0003 (0.007)	0.003 (0.008)	-0.623	-0.4022	6.622	0.002182	C1 < C3<br>C2 < C3
**Entorhinal Cortex L**	-0.074 (0.279)	0.016 (0.282)	0.059 (0.244)	-0.5489	-0.1779	3.474	0.0352	C1 < C3
**Entorhinal Cortex R**	-0.103 (0.195)	0.024 (0.262)	0.080 (0.2484)	-0.7415	-0.2234	8.599	0.000331	C1 < C2<br>C1 < C3
**ParaHippocampal L**	-0.092 (0.216)	0.003 (0.264)	0.1064 (0.218)	-0.9106	-0.4695	9.28	0.000184	C1 < C2<br>C1 < C3<br>C2 < C3
**ParaHippocampal R**	-0.135 (0.239)	0.022 (0.228)	0.122 (0.265)	-0.9707	-0.3781	16.249	6.29E-07	C1 < C2<br>C1 < C3<br>C2 < C3
**Cingulum Post L**	-0.078 (0.129)	0.004 (0.139)	0.087 (0.166)	-0.9985	-0.503	18.559	1.17E-07	C1 < C2<br>C1 < C3<br>C2 < C3
**Cingulum Post R**	-0.100 (0.166)	0.026 (0.154)	0.072 (0.169)	-1.0265	-0.2742	17.744	2.11E-07	C1 < C2<br>C1 < C3
**Retrosplenial Cortex L**	-0.119 (0.151)	0.010 (0.192)	0.126 (0.243)	-1.009	-0.4782	22.126	8.78E-09	C1 < C2<br>C1 < C3<br>C2 < C3
**Retrosplenial Cortex R**	-0.071 (0.126)	0.006 (0.135)	0.076 (0.150)	-0.9776	-0.4635	16.074	7.15E-07	C1 < C2<br>C1 < C3<br>C2 < C3

Mean (SD) of the residuals adjusted for center and ICV, calculated in the overall cohort. These values were used as the input data for the data-driven random forest-based cluster analysis. Additionally, for

plotting purposes in figure 1, the z-score values were calculated for C1 and C2 groups from the residuals adjusted for age, using C3 as the reference group.
Abbreviations: C1 – Cortical predominant subtype; C2 – Fronto-occipital predominant subtype; C3 – Cortical predominant subtype; ICV – intracranial volume; L – Left; R – Right; SD – standard deviation.

Supplementary Table 2. ROIs with the highest contribution to the cluster analysis.

10 TOP ROIs	Gini Index
Right Pallidum	0.9521002
Left frontal inferior operculum	0.9675189
Left Olfactory cortex	0.9774211
Left Pallidum	0.9898548
Left middle cingulum	0.9928215
Pons	0.9928266
Left Caudate	0.9935472
Right Caudate	0.9976649
Right Olfactory cortex	1.0100825
Left Putamen	1.0108899

The 10 ROIs with the lowest Gini Index values. The mean decrease in the Gini index was used to identify the ROIs with the highest contribution to the cluster analysis.

Supplementary Figure 3. Supervised classification tree.

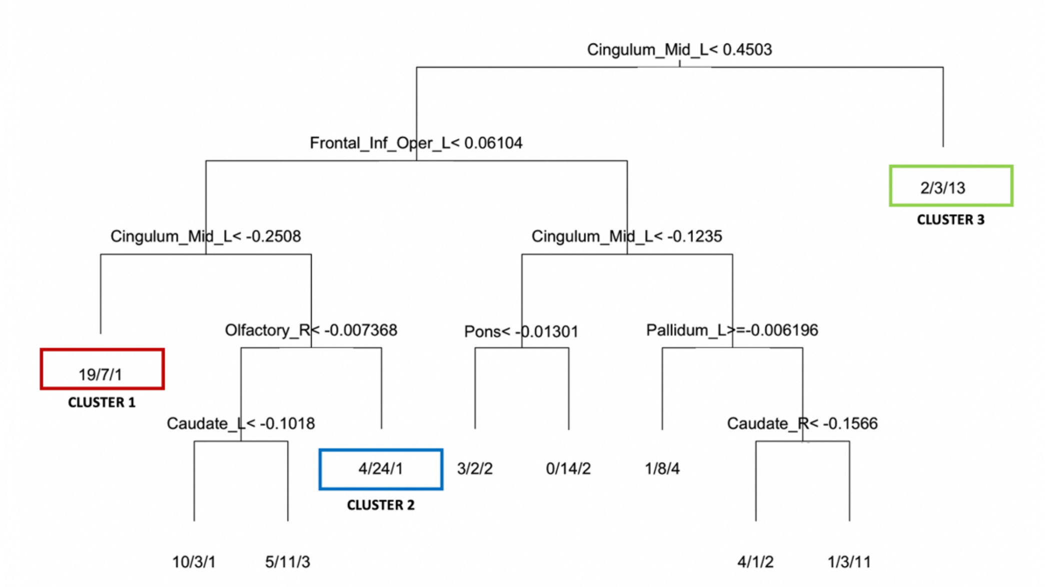

Supervised classification tree showing the complete view of the variable discrimination rules for the DLB clusters. Among the 10 most relevant ROIs, the left middle cingulum, which showed greater volumes in C3 compared to C1 and C2, was placed on top of the tree, indicating that it was the most relevant ROI to discriminate between the DLB clusters. Then, the tree was divided into 2 branches: one that included

C1 and C2, and another one for C3. The branch including C1 and C2 had the left opercular inferior frontal on top of the branch, which had a greater GM volume in C3 compared to C1 and C2, but it did not differ between C1 and C2. Next, the right olfactory cortex was able to discriminate between C1 and C2, with C2 showing larger GM volumes than C1.

Supplementary Table 3. Longitudinal analysis of MMSE trajectories

**MMSE longitudinal analysis adjusted for age**: Mixed effects			
	**Estimate**	**t-stat**	**p-value**
**Intercept**	22.1126	6.479	1.16E-09
**Timepoint 2 (12 months)**	0.01012	0.017	0.98665
**Timepoint 3 (24 months)**	-1.12540	-1.761	0.07972
**Timepoint 4 (36 months)**	-0.95228	-1.083	0.28005
**cluster1**	-3.29427	-2.625	0.00943
**cluster2**	-1.76832	-1.620	0.10697
**Age**	0.03749	0.722	0.47118
**Timepoint 2 – cluster 1 interaction**	-1.60727	-1.905	0.05818
**Timepoint 3 – cluster 1 interaction**	-1.93781	-2.099	0.03702
**Timepoint 4 – cluster 1 interaction**	-3.64796	-2.637	0.00901
**Timepoint 2 – cluster 2 interaction**	-1.01382	-1.273	0.20443
**Timepoint 3 – cluster 2 interaction**	-1.02488	-1.190	0.23552
**Timepoint 4 – cluster 2 interaction**	-2.63218	-2.175	0.03083

**MMSE longitudinal analysis adjusted for age and WMH:** Mixed effects			
	**Estimate**	**t-stat**	**p-value**
**Intercept**	19.99360	5.550	1.24E-07
**Timepoint 2 (12 months)**	-0.01172	-0.019	0.98453
**Timepoint 3 (24 months)**	-1.14797	-1.796	0.07400
**Timepoint 4 (36 months)**	-0.99104	-1.126	0.26136
**Cluster1**	-0.53543	-0.260	0.79541
**Cluster 2**	-0.05834	-0.032	0.97431
**WMH**	0.09485	0.921	0.35837
**Age**	0.05226	0.987	0.32527
**Timepoint 2 – cluster 1 interaction**	-1.60920	-1.907	0.05792
**Timepoint 3 – cluster 1 interaction**	-1.95152	-2.114	0.03576
**Timepoint 4 – cluster 1 interaction**	-3.63543	-2.628	0.00926
**Timepoint 2 – cluster 2 interaction**	-0.98966	-1.243	0.21537
**Timepoint 3 – cluster 2 interaction**	-1.00249	-1.164	0.24585
**Timepoint 4 – cluster 2 interaction**	-2.59825	-2.146	0.03306
**Cluster 1 – WMH interaction**	-0.17555	-1.544	0.12476
**Cluster 2 – WMH interaction**	-0.13685	-1.218	0.22526

Linear mixed effects model consisted of a random intercept per subject with the model reference set to baseline and cluster 3. The outcome was longitudinal MMSE scores (baseline, 12-month, 24-month and 36-month follow-up). The fixed effects were time (categorical), cluster (categorical) and interaction between time and cluster. Abbreviations: Cluster 1 – Cortical predominant subtype; Cluster 2 – Fronto-occipital predominant subtype; Cluster 3 – Subcortical predominant subtype; MMSE – Mini Mental State Examination scores; WMH – White matter hyperintensities.

Supplementary Table 4.

Longitudinal analysis of MMSE trajectories: post-hoc comparisons.

**MMSE longitudinal analysis adjusted for age**								**MMSE longitudinal analysis adjusted for age and WMH**	
**timepoint = MMSE baseline**									
**contrast**	estimate	SE	df	t-stat	p-value	Lower CL	Upper CL	t-stat	p-value
**1-2**	-1.31	0.987	188	-1.33	0.3806	-3.64	1.0196	-1.051	0.5455
**1-3**	-2.79	1.145	188	-2.433	0.0419	-5.49	-0.0805	-2.550	0.0310
**2-3**	-1.47	1.052	188	-1.4	0.3428	-3.96	1.0122	-1.957	0.1259
**timepoint = MMSE 12months**									
**contrast**	estimate	SE	df	t-stat	p-value	Lower CL	Upper CL	t-stat	p-value
**1-2**	-1.95	1.103	260	-1.766	0.1831	-4.55	0.6523	-1.487	0.2991
**1-3**	-4.59	1.234	236	-3.719	0.0007	-7.5	-1.6787	-3.565	0.0013
**2-3**	-2.64	1.148	244	-2.3	0.0577	-5.35	0.0672	-2.625	0.0250
**timepoint = MMSE 24months**									
**contrast**	estimate	SE	df	t-stat	p-value	Lower CL	Upper CL	t-stat	p-value
**1-2**	-2.35	1.173	297	-2.006	0.1124	-5.11	0.4094	-1.674	0.2169
**1-3**	-4.96	1.288	265	-3.847	0.0004	-7.99	-1.9204	-3.681	0.0008
**2-3**	-2.6	1.193	270	-2.182	0.076	-5.42	0.208	-2.548	0.0306
**timepoint = MMSE 36months**									
**contrast**	estimate	SE	df	t-stat	p-value	Lower CL	Upper CL	t-stat	p-value
**1-2**	-2.14	1.551	350	-1.382	0.3517	-5.8	1.5083	-1.336	0.3764
**1-3**	-6.57	1.643	355	-4	0.0002	-10.44	-2.7039	-3.950	0.0003
**2-3**	-4.43	1.46	352	-3.032	0.0073	-7.86	-0.9902	-3.168	0.0048

The method used for degrees of freedom was the Kenward-Roger method, and the p-value adjustment was carried out with the Tukey method for comparing a family of 3 estimates. A confidence level (CL) of 0.95 was used. Abbreviations: MMSE – Mini Mental State Examination scores; WMH – White matter hyperintensities.

Supplementary Table 5. List of ROIs used in the cluster analysis.

LIST OF ROIs ENTERED IN THE ANALYSIS
Precentral bilateral
Frontal Sup bilateral
Frontal Sup Orb bilateral
Frontal Mid bilateral
Frontal Mid Orb bilateral
Frontal Inf Oper bilateral
Frontal Inf Tri bilateral
Frontal Inf Orb bilateral
Rolandic Oper bilateral
Supp Motor Area bilateral
Olfactory bilateral
Frontal Sup Medial bilateral
Frontal Med Orb bilateral
Rectus bilateral
Insula bilateral
Cingulum Ant bilateral
Cingulum Mid bilateral
Hippocampus bilateral
Amygdala bilateral
Calcarine bilateral
Cuneus bilateral
Lingual bilateral
Occipital Sup bilateral
Occipital Mid bilateral
Occipital Inf bilateral
Fusiform bilateral
Postcentral bilateral
Parietal Sup bilateral
Parietal Inf bilateral
SupraMarginal bilateral
Angular bilateral
Precuneus bilateral
Paracentral lobule bilateral
Caudate bilateral
Pallidum bilateral
Putamen bilateral
Thalamus bilateral
Heschl bilateral
Temporal Sup bilateral
Temporal Pole Sup bilateral
Temporal Mid bilateral
Temporal Pole Mid bilateral
Temporal Inf bilateral
Pons
Dorsal Mesopontine
Entorhinal Cortex bilateral
ParaHippocampal bilateral
Cingulum Post bilateral
Retrosplenial Cortex bilateral

Supplementary Methods 1. Longitudinal analysis of MMSE trajectories.

The linear mixed effects model (LMM) was conducted in R version 4.0.3 using the lme4 package. The design consisted of a random intercept per subject with the model reference set to the baseline and cluster 3. The outcome was the longitudinal MMSE scores (12-month, 24-month and 36-month follow-up). The fixed effects were time (categorical), cluster (categorical) and interaction between time and cluster. Post-hoc pairwise comparisons were done between clusters based on model estimates with multiple comparisons corrections with Tukey adjustment (using emmeans package).
