# Supplementary material for: MRI data-driven clustering reveals different subtypes of Dementia with Lewy bodies
Source: NPJ Parkinsons Dis. 2023 Jan 20;9:5. doi: 10.1038/s41531-023-00448-6 (PMC9859778; doi:10.1038/s41531-023-00448-6)
Supplement: Supplementary file 2 — Reporting summary [file 41531_2023_448_MOESM2_ESM.pdf]

nature portfolio

Corresponding author(s): Daniel Ferreira

Last updated by author(s): Aug 30, 2022

Reporting Summary

Nature Portfolio wishes to improve the reproducibility of the work that we publish. This form provides structure for consistency and transparency in reporting. For further information on Nature Portfolio policies, see our Editorial Policies and the Editorial Policy Checklist.

Statistics

For all statistical analyses, confirm that the following items are present in the figure legend, table legend, main text, or Methods section.

n/a	Confirmed	
☐	☒	The exact sample size (*n*) for each experimental group/condition, given as a discrete number and unit of measurement
☐	☒	A statement on whether measurements were taken from distinct samples or whether the same sample was measured repeatedly
☐	☒	The statistical test(s) used AND whether they are one- or two-sided<br>*Only common tests should be described solely by name; describe more complex techniques in the Methods section.*
☐	☒	A description of all covariates tested
☐	☒	A description of any assumptions or corrections, such as tests of normality and adjustment for multiple comparisons
☐	☒	A full description of the statistical parameters including central tendency (e.g. means) or other basic estimates (e.g. regression coefficient) AND variation (e.g. standard deviation) or associated estimates of uncertainty (e.g. confidence intervals)
☒	☐	For null hypothesis testing, the test statistic (e.g. *F*, *t*, *r*) with confidence intervals, effect sizes, degrees of freedom and *P* value noted<br>*Give P values as exact values whenever suitable.*
☒	☐	For Bayesian analysis, information on the choice of priors and Markov chain Monte Carlo settings
☐	☒	For hierarchical and complex designs, identification of the appropriate level for tests and full reporting of outcomes
☒	☐	Estimates of effect sizes (e.g. Cohen's *d*, Pearson's *r*), indicating how they were calculated

Our web collection on statistics for biologists contains articles on many of the points above.

Software and code

Policy information about availability of computer code

Data collection	N/A
Data analysis	SPSS Statistics 27.0;R Foundation for Statistical Computing, version 4.0.3; SPM12 (Wellcome Trust Center for Neuroimaging, London, UK)

For manuscripts utilizing custom algorithms or software that are central to the research but not yet described in published literature, software must be made available to editors and reviewers. We strongly encourage code deposition in a community repository (e.g. GitHub). See the Nature Portfolio guidelines for submitting code & software for further information.

Data

Policy information about availability of data

All manuscripts must include a data availability statement. This statement should provide the following information, where applicable:

- Accession codes, unique identifiers, or web links for publicly available datasets
- A description of any restrictions on data availability
- For clinical datasets or third party data, please ensure that the statement adheres to our policy

The E-DLB consortium and Mayo Clinic make data available to qualified researchers on reasonable request.

Human research participants

Policy information about studies involving human research participants and Sex and Gender in Research.

Reporting on sex and gender	n/a
Population characteristics	The data of this multicenter study were a combination of the E-DLB consortium (n= 97) , including 29 patients from Prague, 34 from Strasbourg, and 34 from Vumc Amsterdam, and the Mayo Clinic DLB cohort from Rochester, MN, United States (n=68), making a total of 165 DLB patients. Diagnosis and presence/absence of core clinical features (parkinsonism, VH, CF and RBD) were based on the 2005 International Consensus Criteria for probable DLB. As a measure of global cognition, we used the Mini-Mental State Examination (MMSE) assessed annually over 3 years.
Recruitment	Please see above.
Ethics oversight	Local ethics committee at each E-DLB center and the Mayo Clinic Institutional Review Board approved the study. Informed consent on participation was obtained from all patients or appropriate surrogates according to the Declaration of Helsinki.

Note that full information on the approval of the study protocol must also be provided in the manuscript.

Field-specific reporting

Please select the one below that is the best fit for your research. If you are not sure, read the appropriate sections before making your selection.

☒ Life sciences ☐ Behavioural & social sciences ☐ Ecological, evolutionary & environmental sciences

For a reference copy of the document with all sections, see nature.com/documents/nr-reporting-summary-flat.pdf

Life sciences study design

All studies must disclose on these points even when the disclosure is negative.

Sample size	A total of 165 probable DLB patients participated in this multicenter study. The data were a combination of the E-DLB consortium (n= 97), including 29 subjects from Prague, 34 from Strasbourg, and 34 from Vumc Amsterdam, and the Mayo Clinic DLB cohort from Rochester, MN, United States (n=68).
Data exclusions	Exclusion criteria were: (i) presence of acute delirium, (ii) terminal illness, (iii) previous stroke, (iv) psychotic or bipolar disorder, (v) craniocerebral trauma, and (vi) recent diagnosis of a major somatic illness.
Replication	To carefully test the robustness and stability of our cluster analysis, we carried out a random forest proximity matrix assessment. In this analysis we assessed the stability of the similarity matrix – the input of our clustering – by repeating the random forest 100 times. We computed the difference between the similarity matrix used in the main analysis and each of the 100 simulated similarity matrices obtained from the 100 random forests.
Randomization	We adjusted for total intracranial volume and centre of origin before performing the clustering. We also adjusted for age in the post-hoc analyses.
Blinding	Blinding was not necessary as no intervention was carried out.

Reporting for specific materials, systems and methods

We require information from authors about some types of materials, experimental systems and methods used in many studies. Here, indicate whether each material, system or method listed is relevant to your study. If you are not sure if a list item applies to your research, read the appropriate section before selecting a response.

Materials & experimental systems

n/a	Involved in the study
☒	☐ Antibodies
☒	☐ Eukaryotic cell lines
☒	☐ Palaeontology and archaeology
☒	☐ Animals and other organisms
☒	☐ Clinical data
☒	☐ Dual use research of concern

Methods

n/a	Involved in the study
☒	☐ ChIP-seq
☒	☐ Flow cytometry
☐	☒ MRI-based neuroimaging

Magnetic resonance imaging

Experimental design

Design type	Structural MRI T1-based study.
Design specifications	n/a
Behavioral performance measures	n/a

Acquisition

Imaging type(s)	Structural
Field strength	1.5T and 3T
Sequence & imaging parameters	CMRR, Strasbourg: Siemens Verio 3T; T1-weighted: TR=1900, TE=2.53.<br>Mayo Clinic: Discovery 750MR 3T; T1-weighted: TR=2300, TE=3.<br>Motol University Hospital, Prague: Siemens Avanto 1.5T; T1-weighted: TR=2000, TE=3.08.<br>VUmc, Amsterdam: Discovery 750MR; T1-weighted: TR=8, TE=3.
Area of acquisition	Whole brain

Diffusion MRI ☐ Used ☒ Not used

Preprocessing

Preprocessing software

Images from the E-DLB consortium were managed through the HiveDB database system (1). All the data was preprocessed at the Mayo Clinic. Using ANTs (2), the Mayo Clinic Adult Lifespan Template (MCALT) (https://www.nitrc.org/projects/mcalt/) atlas was propagated to individuals' native MPRAGE space and regional estimations of volume across cortical and subcortical GM structures were calculated. Tissue probabilities were determined for each MPRAGE using the unified segmentation algorithm in SPM12 (Wellcome Trust Center for Neuroimaging, London, UK), with MCALT tissue priors and settings (3). A total of 82 cortical ROIs, 12 subcortical ROIs and 2 brainstem ROIs were used in this study. While estimations of both volume and thickness can be obtained for cortical areas, in this study we used volume estimations for all the ROIs (cortical and subcortical), to avoid combination of measures in different scales, which can influence the results in cluster analysis. The total intracranial volume (ICV) was calculated from the tissue probabilities.

References:

1. Muehlboeck, J. S., Westman, E. & Simmons, A. TheHiveDB image data management and analysis framework. Front. Neuroinform. 7, 1–13 (2014).
2. Avants, B. B., Epstein, C. L., Grossman, M. & Gee, J. C. Symmetric diffeomorphic image registration with cross-correlation: Evaluating automated labeling of elderly and neurodegenerative brain. Med. Image Anal. 12, 26–41 (2008).
3. Schwarz, C. G. et al. [P2-415]: the Mayo Clinic Adult Lifespan Template: Better Quantification Across the Lifespan. Alzheimer's Dement. 13, P792–P792 (2017).

Normalization	Specifications above (preprocessing software section).
Normalization template	Specifications above (preprocessing software section).
Noise and artifact removal	Specifications above (preprocessing software section).
Volume censoring	Specifications above (preprocessing software section).

Statistical modeling & inference

Model type and settings

Differences in demographic and clinical measures as well as in biomarkers were assessed with one-way ANOVA for continuous variables and the Pearson's chi-square test for categorical variables. Differences between GM across ROIs were assessed with ANCOVA adjusting by age. These analyses were performed using IBM SPSS Statistics 27.0 (IBM Corp., Armonk, New York). The results from ANCOVA were corrected for multiple comparisons using the false-discovery rate (FDR) adjustment across the 96 ROIs, with the significance level set at $p < 0.05$. A linear mixed model (LMM) was implemented in R to assess cognitive decline over 3 years as measured by MMSE scores. The LMM was rerun accounting for concomitant AD pathology, WMH burden, and APOE genotype, to test for the independent effect of DLB subtype in the context of the other potential modifiers of MMSE trajectories. All analyses in this study are cross-sectional except for the LMM for MMSE scores, which had a repeated measures design.

Effect(s) tested	Please see "Model type and settings" for details.

Specify type of analysis: ☒ Whole brain ☐ ROI-based ☐ Both

Statistic type for inference
(See Eklund et al. 2016)

Specify voxel-wise or cluster-wise and report all relevant parameters for cluster-wise methods.

Correction

The results from ANCOVA were corrected for multiple comparisons using the false-discovery rate (FDR) adjustment across the 96 ROIs.

Models & analysis

n/a	Involved in the study
☒	☐ Functional and/or effective connectivity
☒	☐ Graph analysis
☐	☒ Multivariate modeling or predictive analysis

Multivariate modeling and predictive analysis

To account for the potential confounding effect of scanner variability across centers, as well as between-subject variability in head size, a multiple linear regression model was fitted per ROI (outcome variable), with center and ICV included as predictors.
